# Orthostatic Hypotension Is Associated With Cognitive Decline in Parkinson Disease

**DOI:** 10.3389/fneur.2020.00897

**Published:** 2020-09-02

**Authors:** Katherine Longardner, Ece Bayram, Irene Litvan

**Affiliations:** Department of Neurosciences, UC San Diego Health System, University of California, San Diego, La Jolla, CA, United States

**Keywords:** orthostatic hypotension, Parkinson disease, cognition, dysautonomia, movement disorders

## Abstract

**Introduction:** Cognitive impairment and orthostatic hypotension (OH) are common, disabling Parkinson disease (PD) symptoms that are strongly correlated. Whether the relationship is causative or associative remains unknown. OH may occur without classic orthostatic symptoms of cerebral hypoperfusion (i.e., lightheadedness or dizziness). Whether longitudinal differences in cognition occur between symptomatic and asymptomatic OH patients has not been explored.

**Objectives:** We characterized the prevalence of OH, orthostatic symptoms, and cognitive impairment among PD patients and compared cognition between patients with and without OH, and between patients with symptomatic and asymptomatic OH.

**Methods:** Our cross-sectional, retrospective, observational study included 226 clinically diagnosed PD patients who underwent repeated standardized evaluations. Among these, 62 had longitudinal follow-up of > 3.5 years. We compared longitudinal Montreal Cognitive Assessment (MoCA) scores between patients remaining OH-free (*n* = 14) and those without baseline OH that developed OH (*n* = 28), matched for age, sex, education, and PD duration. We also compared MoCA scores between groups with asymptomatic OH (*n* = 13) and symptomatic OH (*n* = 13) matched for the same factors.

**Results:** In the cross-sectional analysis, OH patients had worse cognition. In the longitudinal analysis (mean follow-up = 5.3 years), OH patients had worse cognitive decline (*p* = 0.027). Cognitive impairment was similar between asymptomatic and symptomatic OH patients in the cross-sectional and longitudinal analyses.

**Conclusions:** OH is associated with cognitive impairment in PD. Further studies are needed in larger cohorts to expand our findings and to determine whether treating OH can prevent or delay cognitive dysfunction.

## Introduction

While the manifestations of Parkinson disease (PD) affecting movement are well-recognized, PD also causes myriad non-motor symptoms, including cognitive and autonomic disorders, which can be as disabling as motor symptoms (1). Approximately 25% of PD patients have cognitive dysfunction at any given time (2). The likelihood of developing cognitive impairment increases with disease duration—up to 50% of cognitively normal individuals develop mild cognitive impairment within 6 years of PD onset, and over 80% develop dementia within 20 years (3, 4).

Autonomic nervous system dysfunction causing neurogenic orthostatic hypotension (OH) affects up to half of PD patients (5). OH is defined as a drop in systolic BP (SBP) of at least 20 mmHg or diastolic BP (DBP) of at least 10 mmHg within 3 min of standing (6). OH may manifest with temporary symptoms caused by hypoperfusion to the brain and other organs when upright, including lightheadedness, fatigue, dizziness, syncope, and visual, gait, and cognitive disturbances. Orthostatic symptoms increase functional disability and fall risk and negatively affect quality of life (7, 8). Cognitive impairment and OH are strongly correlated in PD, although the underlying pathophysiology remains unclear (9, 10). Potential contributing factors include neurodegeneration, repeated episodes of cerebral hypoperfusion, and/or noradrenergic deficits (9–11).

Therapeutic strategies for OH aim to raise blood pressure (BP) to reduce problematic orthostatic symptoms related to hypoperfusion (i.e., feeling lightheaded, dizzy, or faint when standing, or syncope). Treatment options include non-pharmacologic measures such as increasing hydration, consuming extra sodium, and using an abdominal binder use, as well as adding pharmacological agents including droxidopa, midodrine, fludrocortisone, and/or pyridostigmine (6). However, OH treatment is complex; orthostatic symptoms are often vague and non-specific, and may be difficult to distinguish clinically from other levodopa-related fluctuating symptoms in parkinsonian patients. In patients with neurogenic OH, autonomic dysfunction frequently causes concomitant supine hypertension (SH), which further complicates treatment given the potential risks of acute cardiovascular problems related to hypertension (12). Among PD patients, SH is also associated with worse cognition (13). However, whether long-term hypotension or hypertension is worse for cognition in PD remains to be explored. Generally, the urgency of increasing BP to prevent injuries associated with syncope and falls related to OH outweighs the risk of exacerbating SH (14).

Although the decision whether to treat OH is typically based on whether orthostatic symptoms are present (e.g., lightheadedness, dizziness), OH can occur without symptoms. The clinical relevance of asymptomatic OH (aOH) is unknown (15). Orthostatic symptoms may not correlate with absolute BP or the magnitude of BP drop (16). Additionally, basing the decision to treat OH solely on patient-reported symptoms when standing may miss individuals with unrecognized orthostatic cognitive fluctuations, which can occur without overt symptoms (17, 18). Currently, no therapeutic guidelines exist regarding whether to treat only OH patients suffering from symptoms when upright, or to treat a hemodynamic target. Although limited research exists comparing aOH and symptomatic OH (sOH) in PD, studies suggest similar ambulatory and functional capacity, falls, and health care utilization across both groups (15, 19). Thus, allowing repeated asymptomatic cerebral hypoperfusion to go untreated might cause worsening cognition over time. Alternatively, OH and cognitive impairment may be associated for other reasons. Whether aOH and sOH patients have longitudinal cognitive differences remains unknown. Several studies evaluating the relationship between OH and cognition longitudinally among PD patients found that OH is associated with cognitive decline (20, 21), but did not distinguish between aOH and sOH. A better understanding of the relationship between OH and cognitive impairment, and of the clinical significance of OH symptoms is essential to guide therapeutic decision-making.

This retrospective observational study aimed to investigate the relationship between OH, orthostatic symptoms, and cognition among patients with clinically defined PD seen at the University of California San Diego (UCSD) Movement Disorders Center. We aimed to (1) characterize the prevalence of OH, orthostatic symptoms, SH, and cognitive impairment in our cohort using cross-sectional data; and (2) compare change in Montreal Cognitive Assessment (MoCA) (22) scores over time between PD patients with OH (OH+) and without OH (OH–), and between those with aOH and sOH using longitudinal data. Based on existing literature and clinical experience, we hypothesized that OH would be associated with cognitive decline and that cognitive impairment would be similar between aOH and sOH patients.

## Methods and Materials

### Participants

Data were collected from patients seen by one movement disorders specialist (Dr. IL) at University of California San Diego Parkinson and Other Movement Disorders Center outpatient clinic between December 2011 and March 2020 under an Institutional Review Board-approved clinical research database. All patients provided written informed consent. Only patients with a clinical diagnosis of PD based on Movement Disorders Society (MDS) Clinical Diagnostic Criteria (23) were included. Exclusion criteria were as follows: (1) clinical findings consistent with atypical parkinsonism (including cognitive impairment within 1 year of motor symptom onset suggestive of dementia with Lewy bodies, severe and early autonomic failure suggestive of multiple system atrophy, etc.), (2) unclear diagnosis due to confounding medical conditions and/or an imprecise timeline, and (3) secondary parkinsonism, including normal pressure hydrocephalus, vascular parkinsonism, drug-induced parkinsonism, and fragile-X associated tremor/ataxia syndrome.

For the cross-sectional analysis (*n* = 226), the first clinic visit with complete data was selected. In most patients, this was the first visit, but if baseline visit data were incomplete (e.g., only one set of vital signs, missing MoCA, etc.), the subsequent chronologic visit with complete data was chosen. Patients returned for follow-up visits at clinically indicated intervals typically ranging between 6 and 12 months, and were evaluated by the same movement disorders specialist.

**Figure 1:**
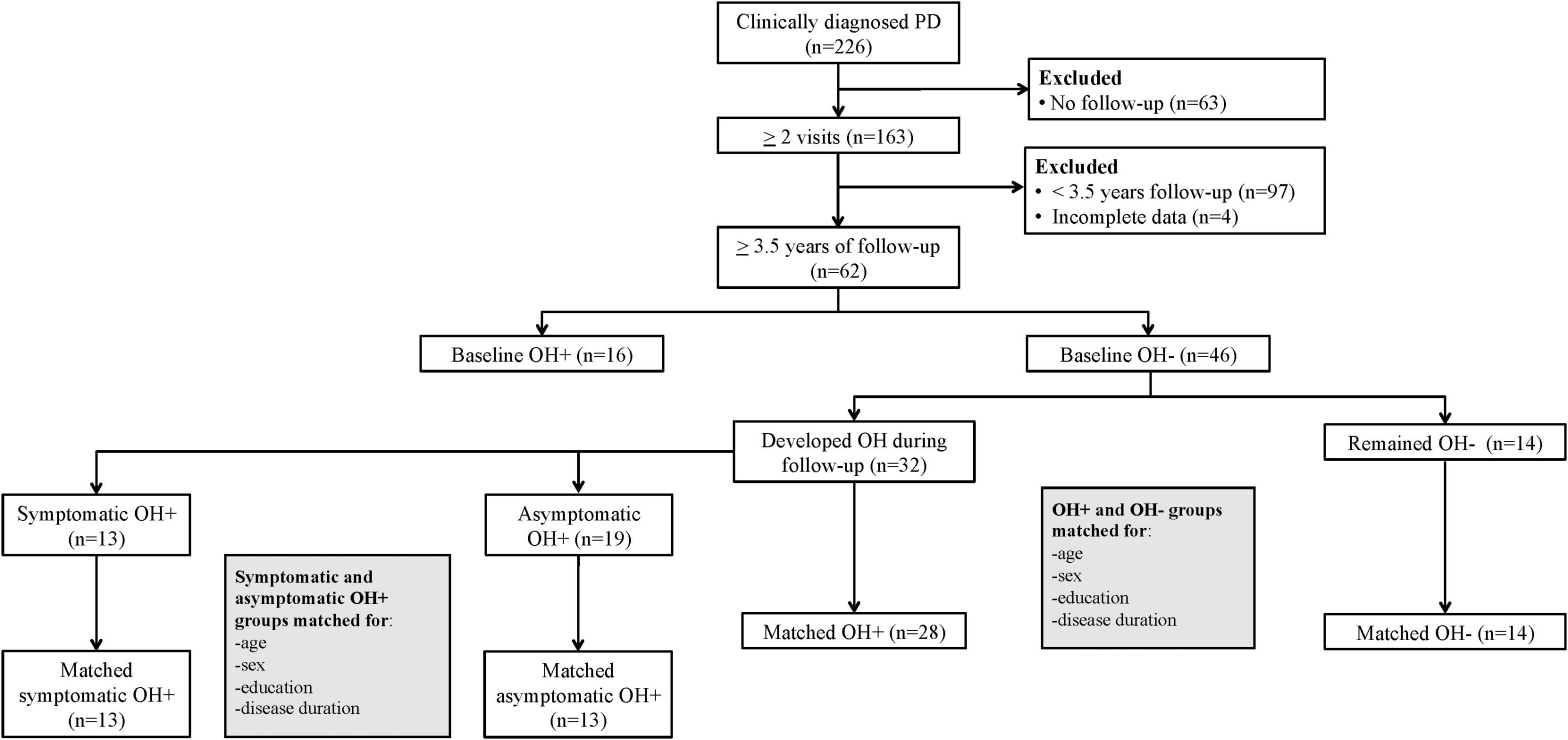
Flow diagram of Parkinson disease (PD) patients with orthostatic hypotension (OH+) and without OH (OH–) included in the longitudinal comparisons.

For the longitudinal analysis, we included only PD patients with at least 3.5 years of follow-up; patients with incomplete data were excluded. This interval was selected based on prior research in PD patients that showed medium to large effect sizes for cognitive changes with follow-up testing at 4 years (24), and no significant MoCA score change after 3 years (25), supporting a longer study duration. Additionally, among a community-based sample of older adults, repeated MoCA was able to detect cognitive changes over a 3.5-year period (26). Among the 62 patients with a minimum 3.5-year follow-up interval, 16 had OH during the initial visit, 32 developed OH during follow-up, and 14 remained OH– (Figure 1). To evaluate OH group differences for MoCA change over time, the group that remained OH– (*n* = 14) was matched with the OH+ group (including only the patients without OH at the initial visit that developed OH during follow-up) for baseline age, sex, education, and disease duration (*n* = 28). Among the 32 patients that developed OH, 13 reported orthostatic symptoms at the initial visit that OH was diagnosed (sOH), while 19 did not (aOH). To evaluate sOH and aOH group differences for MoCA change over time, the sOH group (*n* = 13) was matched with the aOH group (*n* = 13) for baseline age, sex, education, and disease duration.

### Clinical Evaluations

#### BP Measurements

Medical staff routinely measured orthostatic vital signs during each clinic visit using an electronic inflatable brachial sphygmomanometer after several minutes supine, 1 min after standing, and 3 min after standing. OH was defined as at least 20 mmHg drop in SBP and/or at least 10 mmHg drop in DBP within 3 min of standing (6). SH was defined as SBP at least 140 mmHg or DBP at least 90 mmHg while supine (12).

#### Rating Scales

At each clinic visit, patients were evaluated using the MoCA (22), the MDS-Unified Parkinson Disease Rating Scale (MDS-UPDRS) (27), and the Hoehn and Yahr (H&Y) Scale (28). The MoCA, a brief multi-domain cognitive screening test (maximum score is 30, higher is better), was administered using different test versions at subsequent visits to minimize learning effects. Mild cognitive impairment (MCI) was defined by a total MoCA score cutoff <26, which has 90% sensitivity and 75% specificity for PD-MCI (29), and at least 21. PD-dementia was defined as a total MoCA score <21, which has 81% sensitivity and 95% specificity (29).

The patient and/or caregiver completed the MDS-UPDRS Part 1 and 2 questionnaires. The same movement disorders specialist reviewed the questionnaire responses and performed the MDS-UPDRS Part 3 and H&Y scale at each visit. MDS-UPDRS Part 1 Item 12 (1.12, Lightheadedness on Standing: “Over the past week, have you felt faint, dizzy, or foggy when you stand up after sitting or lying down?”) was used to categorize OH+ patients as aOH and sOH. We defined aOH as presence of OH with an Item 1.12 score of 0, and defined sOH as presence of OH with Item 1.12 score > 0. The movement disorders specialist verbally confirmed with all patients that the response to this item referred to the sensation of fainting when upright rather than postural instability in order to reduce the likelihood of false positives.

### Statistics

Statistical analyses were performed using IBM SPSS v27 (IBM Corp., Armonk, N.Y., USA). For cross-sectional analyses, Fisher's exact, independent *t*, and Mann–Whitney *U* tests were used. MoCA between groups were compared using general linear model including age and disease duration, which differed for OH+ and OH– groups, as covariates. Bonferroni correction was used for multiple comparisons. Longitudinal analyses were conducted using individual linear mixed models with autoregressive order 1 covariance structure. Models included main effect of groups (either OH+ vs. OH– or sOH vs. aOH), interaction of groups with time (interval between baseline MoCA and each longitudinal MoCA score), and baseline MoCA as a covariate. The same analyses were also performed for MDS-UPDRS Part 3 scores for the groups, with baseline MDS-UPDRS Part 3 as a covariate. Cohen's *f* ^2^ was estimated for effect sizes in the models (30); a value of 0.02 indicates a small effect, 0.15 indicates a medium effect and 0.35 indicates a large effect (31). *p* <0.05 was considered statistically significant.

## Results

### Cross-Sectional Analyses

Among our 226 PD patients (34.1% women), 69 (30.5%) had OH. Among these 69 OH+ patients, 45 (65.2%) were asymptomatic. About one-third (*n* = 73) of all patients had PD-MCI, and 11.9% (*n* = 27) had PD-dementia. Compared to OH– patients, OH+ patients were older and had longer disease duration, worse motor symptom severity, more levodopa use, and lower MoCA scores (Table 1). In the model adjusted for age and disease duration, MoCA was lower in OH+ compared to the OH– group, although this difference remained at a trend level [OH– mean (standard error, SE) = 25.3 (0.3) vs. OH+ mean (SE) = 24.2 (0.5); *F*_(1, 222)_ = 3.53, *p* = 0.062, $\eta_{\text{p}}^{2}$ = 0.016]. There were no differences in demographics, clinical features, mean BP change, or MoCA scores between aOH and sOH patients. Clinical and demographic characteristics were also similar between OH+ patients with and without SH (Supplementary Table 1).

**Table 1:** Cross-sectional comparison of clinical characteristics, demographics, and cognition between Parkinson disease patients with and without orthostatic hypotension (OH) and between patients with symptomatic OH and asymptomatic OH.

	**OH– (*n* = 157)**	**OH+ (*n* = 69)**	***p*-value for OH– vs. OH+**	**aOH^**a**^ (*n* = 45)**	**sOH^**a**^ (*n* = 24)**
Age, years	64.8 (10.8)	71.0 (9.3)	<0.001*	71.3 (9.7)	69.2 (9.1)
Sex, female (%)	50 (31.8)	27 (39.1)	1.00	16 (35.6)	11 (45.8)
Education, years^b^	16.3 (2.8)	16.3 (3.3)	1.00	16.9 (3.4)	15.6 (3.1)
Disease duration, years	4.5 (4.0)	6.9 (4.9)	0.002*	7.0 (4.9)	6.7 (4.9)
Levodopa use (%)	74 (47.1)	48 (69.5)	0.029*	32 (71.1)	16 (66.7)
Supine hypertension (%)	29 (18.5)	40 (58.0)	<0.001*	24 (53.3)	16 (66.7)
SBP change from supine to standing at 3 min, mmHg	0.0 (9.6)	−23.6 (14.0)	<0.001*	−24.6 (13.5)	−21.7 (15.3)
DBP change from supine to standing at 3 min, mmHg	+4.8 (6.8)	−4.5 (9.3)	<0.001*	−4.7 (9.6)	−4.3 (8.7)
Hoehn & Yahr scale	2.1 (0.7)	2.4 (0.8)	0.024*	2.4 (0.9)	2.5 (0.8)
MDS-UPDRS Part 3	25.6 (12.9)	30.6 (16.0)	0.18	29.8 (15.0)	31.9 (17.9)
MoCA score	25.5 (3.3)	23.7 (5.0)	0.016*	24.0 (4.9)	23.0 (5.2)
MCI (%)	50 (31.8)	23 (33.3)	1.00	13 (28.9)	10 (41.7)
Dementia (%)	12 (7.6)	15 (21.7)	0.081	10 (22.0)	5 (20.8)

Continuous variables are reported as mean (standard deviation); categorical variables are reported as number (percentage). Statistical significance marked with *. The results reported in this table are the results of Fisher's exact test and independent t-test, with Bonferroni-adjusted p-values.

a *All Bonferroni-adjusted p-values are 1.00 for aOH vs. sOH comparisons*.

b *Data missing for 55 patients*.

*OH, orthostatic hypotension; OH–, without OH; OH+, with OH; aOH, asymptomatic OH; sOH, symptomatic OH; SBP, systolic blood pressure; DBP, diastolic blood pressure; MDS-UPDRS, Movement Disorders Society Unified Parkinson Disease Rating Scale; MoCA, Montreal Cognitive Assessment; MCI, mild cognitive impairment*.

*Statistically significant values are in bold*.

### Longitudinal Analyses

Among the 226 patients assessed, 164 were excluded from the longitudinal analysis due to <3.5 years follow-up or missing data (Figure 1). Those excluded were older, with longer disease duration, and worse motor symptom severity (Supplementary Table 2). In the 62 subjects with minimum 3.5 years of follow-up, the mean follow-up interval was 5.3 (±1.3) years.

**Figure 2:**
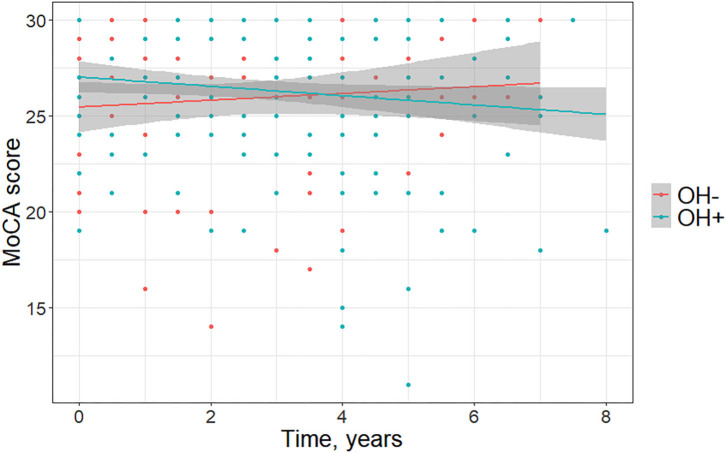
Change in Montreal Cognitive Assessment (MoCA) scores over time in Parkinson disease patients without orthostatic hypotension (OH–) and with orthostatic hypotension (OH+) (graph depicts least square regression lines and 95% confidence intervals).

Demographics and clinical characteristics of groups included in the longitudinal analyses are summarized in Table 2. For the longitudinal model including the OH– (*n* = 14) and the OH+ groups (*n* = 28), OH did not have a main effect on MoCA [*F*_(1, 228)_ = 3.31, *p* = 0.070, *f* ^2^ = 0.015] or MDS-UPDRS Part 3 [*F*_(1, 228)_ = 0.14, *p* = 0.71, *f* ^2^ = 0.0006]. MoCA score declined more for the OH+ group over time [*F*_(2, 228)_ = 3.67, *p* = 0.027, *f* ^2^ = 0.032] (Figure 2). MDS-UPDRS Part 3 score increased more for the OH– group over time [*F*_(2, 228)_ = 4.62, *p* = 0.011, *f* ^2^ = 0.041].

**Table 2:** Comparison of demographic and clinical characteristics between Parkinson disease patients that remained without OH during the follow-up period (OH–) and patients without OH at baseline that developed OH during the follow-up period; and asymptomatic and symptomatic OH patients within the OH+ group based on initial visit with OH.

	**OH– patients (*n* = 14)**	**OH+ patients (*n* = 28)**	***p*-value for OH– vs. OH+**	**aOH patients (*n* = 13)**	**sOH patients (*n* = 13)**	***p*-value for aOH vs. sOH**
Baseline age, years	58.3 (11.0)	62.7 (11.0)	0.22	64.1 (14.0)	63.2 (7.4)	0.72
Sex, female (%)	4 (28.6)	9 (32.1)	1.00	5 (38.5)	4 (30.8)	1.00
Education, years	15.1 (3.3)	15.8 (3.2)	0.41	16.0 (2.4)	15.2 (3.7)	0.73
Baseline disease duration, years	2.4 (1.9)	3.2 (2.4)	0.39	3.1 (1.8)	4.5 (2.7)	0.20
Follow-up interval, years	5.0 (1.1)	5.5 (1.3)	0.26	5.7 (1.0)	5.3 (1.5)	0.28
Baseline Hoehn & Yahr scale	1.6 (0.6)	1.8 (0.5)	0.45	1.8 (0.6)	2.0 (0.4)	0.40
Follow-up Hoehn & Yahr scale	2.1 (0.7)	2.3 (0.6)	0.55	2.2 (0.6)	2.4 (0.7)	0.43
Baseline MDS-UPDRS Part 3	21.1 (15.4)	21.0 (9.4)	0.61	23.2 (10.5)	22.6 (8.6)	0.82
Follow-up MDS-UPDRS Part 3	25.1 (15.5)	21.8 (12.7)	0.59	19.9 (11.1)	22.5 (12.6)	0.49
Baseline MoCA	25.8 (3.1)	26.1 (2.4)	0.97	25.8 (2.1)	25.1 (2.6)	0.66
Follow-up MoCA	25.6 (4.1)	25.0 (4.7)	0.74	26.9 (3.0)	25.1 (3.9)	0.22

*All variables are reported as mean (standard deviation) or percentage. Follow-up visits indicate last visits during follow-up interval. The results reported in this table are the results of Fisher's exact test and Mann–Whitney U-test*.

*OH, orthostatic hypotension; OH–, without OH; OH+, with OH; aOH, asymptomatic OH; sOH, symptomatic OH; MDS-UPDRS, Movement Disorders Society Unified Parkinson Disease Rating Scale; MoCA, Montreal Cognitive Assessment*.

For the longitudinal model including aOH (*n* = 13) and sOH groups (*n* = 13), orthostatic symptom presence did not have a main effect on MoCA [*F*_(1, 147)_ = 0.039, *p* = 0.85, *f* ^2^ = 0.0003] or MDS-UPDRS Part 3 [*F*_(1, 144)_ = 0.047, *p* = 0.83, *f* ^2^ = 0.0003]. There were also no orthostatic symptom presence and time interaction for MoCA or MDS-UPDRS Part 3 [*F*_(2, 147)_ = 1.38, *p* = 0.25, *f* ^2^ = 0.019; *F*_(2, 144)_ = 1.85, *p* = 0.16, *f* ^2^ = 0.026]. Baseline MoCA/MDS-UPDRS Part 3 scores were associated with longitudinal MoCA/MDS-UPDRS Part 3 scores for all models (*p* <0.001 for all, *f* ^2^ > 0.45 for MoCA, *f* ^2^ > 0.21 for MDS-UPDRS Part 3 models).

## Discussion

The cross-sectional analysis of 226 patients showed that OH+ patients had worse total MoCA scores. After adjusting for age and disease duration, this difference remained at a trend level. Our longitudinal analysis comparing MoCA performance in 42 PD patients with and without OH matched for age, education, sex, and disease duration found worse cognitive decline among OH+ patients.

We found no cross-sectional or longitudinal differences in cognition between PD patients with aOH and sOH, although the group sizes in the longitudinal comparisons were small. These findings warrant further research regarding whether cognitive decline varies between OH patients with and without orthostatic symptoms. To date, only one cross-sectional study has explored cognitive differences between PD patients with aOH and sOH (15). This research found no difference in MoCA scores between aOH and sOH or between OH– and OH+, substantiating the need for longitudinal cognitive assessment to better understand OH's role in cognitive decline. Our results reinforce the limited studies showing that patients with aOH and sOH have similar clinical features (15, 19), which supports the importance of OH screening in PD irrespective of whether patients report classic orthostatic symptoms (i.e., lightheadedness, dizziness, or fogginess on standing), especially since two-thirds of our cohort were asymptomatic. Therefore, in clinical practice, we advocate routinely measuring orthostatic vital signs while supine, 1 min after standing, and 3 min after standing for all PD patients (6). We also recommend implementing this approach to address OH in large prospective PD research studies.

Given the retrospective design, our study cannot determine whether OH directly contributed to cognitive deterioration or whether it is simply associated. Additional research is needed to clarify whether a causal relationship exists between OH and chronic cognitive impairment. Several studies have correlated acute hypotensive episodes with temporary cognitive worsening in PD, even in individuals with normal baseline cognition (17, 18, 32). Episodic OH likely transiently affects cognition by changing regional cerebral blood flow patterns (33). However, the mechanism of how OH affects the brain over time in PD is uncertain. OH may negatively affect cognition through hypoperfusion that directly and reversibly induces cortical dysfunction, or may cause cumulative brain damage from either oxidative stress, accelerated neurodegeneration, or microvascular insult (34, 35). Alternatively, OH may be a marker for a PD subtype that progresses more rapidly or causes cognitive decline for other reasons. Concurrent SH related to dysautonomia may also contribute to cognitive impairment in PD (13), although we did not find cross-sectional differences in cognition between OH+ patients with and without SH in our population.

Currently, no prevention exists for cognitive deterioration in PD (36). If OH represents a potential modifiable risk factor for cognitive impairment, then diagnosing and treating OH early would be an important strategy to reduce the risk of imminent cognitive decline. Several studies have examined whether anti-hypotensive medications benefit cognition in OH+ patients. Among 10 OH+ spinal cord injury patients, treating hypotension with midodrine improved verbal fluency compared to age- and sex-matched controls (33). After initiating midodrine, a PD-dementia patient with severe OH had sustained improvement in cognition and hypotensive episodes (37). A cohort of 40 OH+ parkinsonian patients showed better Cognitive Functional Independence Measures following treatment with midodrine and/or fludrocortisone (38). Although these results are suggestive, prospective studies with greater sample sizes are needed to establish whether treating OH can delay or prevent cognitive decline in PD.

Despite being retrospective, a strength of this study is the prospective standardized evaluation that included orthostatic BP measurements, MoCA repeated every 6 months, and longitudinal MDS-UPDRS evaluations performed by the same rater. Our study has several limitations. The main limitation is the small sample size due to the retrospective methodology. Due to the retrospective and observational nature of the study, all participants had follow-up visits at different time points, leading to missing data for some time points. While we only included those with over 3.5 years of follow-up in our longitudinal analyses, the non-significant group differences and small effect sizes suggest that this sample did not have pronounced differences in terms of cognitive decline. A more systematic assessment of cognitive decline in this population is necessary to draw reliable conclusions.

Another limitation is the use of MoCA as an outcome for cognition. Although relatively rapid, easy to administer in the office setting, and sensitive for diagnosing cognitive impairment in PD (29), MoCA was impractical to track cognitive decline in PD over 3 years (25), and does not allow detailed examination of individual cognitive domains. In our study, the mean MoCA score changed minimally in PD patients over the mean 5.3-year follow-up. Despite rotating different MoCA test versions at follow-up visits, there may have been learning effects. More extensive neuropsychological testing would be more sensitive to evaluate cognitive changes over time, and provide more reliable information on individual cognitive domains.

Although we evaluated for OH at each visit, in-office orthostatic BP measurements may not reflect the severity of OH occurring throughout the day in up to two-thirds of patients with chronic autonomic failure and may miss patients with delayed OH (39). Future studies to detect the impact of OH on cognition should use ambulatory BP testing, which is a more sensitive measure. Additionally, MDS-UPDRS Item 1.12 has not been validated for sOH. Since specific autonomic testing was not performed (e.g., beat-by-beat BP monitoring with Valsalva maneuver or plasma norepinephrine levels), we cannot be certain that all OH+ patients had neurogenic OH. Thus, some patients with secondary OH may have been included. However, the etiology of OH would unlikely impact our findings. Furthermore, our sample consisted of only PD patients that consented to participate in clinical research, so our findings may not be generalizable to all persons with PD. There were clinical differences between the population included in the longitudinal analysis and those excluded due to <3.5 years of follow-up: patients included were younger, had shorter disease duration, and milder motor symptoms. These differences may be due to the fact that the older, sicker patients had difficulty attending appointments or they deceased during the study period.

To conclude, our findings support prior research demonstrating a strong relationship between OH and cognitive impairment in PD (9, 10), and add to the limited literature investigating clinical differences between patients with aOH and sOH, corroborating the similarities between these groups. While additional research with a greater sample size is needed to expound our findings, if OH contributes to cognitive impairment rather than merely being associated, it would be pertinent to identify and treat OH early as a modifiable risk factor for cognitive impairment in PD. Larger prospective longitudinal studies with comprehensive cognitive testing are warranted to determine whether treating OH in PD can prevent or delay cognitive decline, given the important implications for clinical practice.

## Data Availability Statement

The raw data supporting the conclusions of this article will be made available by the authors, without undue reservation.

## Ethics Statement

The studies involving human participants were reviewed and approved by University of California San Diego Institutional Review Board. The patients/participants provided their written informed consent to participate in this study.

## Author Contributions

KL: collected and organized the data, drafted the manuscript, and contributed to statistical analysis. EB: contributed to statistical analysis and critically reviewed the manuscript. IL: conceptualized the study and critically reviewed the manuscript. All authors contributed to the article and approved the submitted version.

## Conflict of Interest

The authors declare that the research was conducted in the absence of any commercial or financial relationships that could be construed as a potential conflict of interest.

## Abbreviations

PD: Parkinson disease
OH: Orthostatic hypotension
OH–: Without orthostatic hypotension
OH+: With orthostatic hypotension
aOH: Asymptomatic orthostatic hypotension
sOH: Symptomatic orthostatic hypotension
SH: Supine hypertension
MoCA: Montreal Cognitive Assessment
MDS: Movement Disorders Society
MDS-UPDRS: Movement Disorders Society Unified Parkinson Disease Rating Scale
BP: Blood pressure
SBP: Systolic blood pressure
DBP: Diastolic blood pressure
H&Y: Hoehn and Yahr
MCI: Mild cognitive impairment.
