# Supplementary material for: Orthostatic Hypotension Is Associated With Cognitive Decline in Parkinson Disease
Source: Front Neurol. 2020 Sep 2;11:897. doi: 10.3389/fneur.2020.00897 (PMC7492200; doi:10.3389/fneur.2020.00897)
Supplement: Supplementary file 1 [file Table_1.docx]

**Supplementary Table 1.** Cross-sectional comparison of clinical characteristics, demographics, and cognition in Parkinson disease patients with orthostatic hypotension (OH) without supine hypertension (OH+SH-) and with OH and supine hypertension (OH+SH+).

|  | **OH+SH- (n=29)** | **OH+SH+ (n=40)** | ***p* value for OH+SH- vs OH+SH+** |
| --- | --- | --- | --- |
| **Age, years (SD)** | 69.4 (10.2) | 72.1 (8.9) | 1.00 |
| **Sex, female (% )** | 13 (44.8) | 14 (35.0) | 1.00 |
| **Education, years (SD)**  *(Data missing for 21.7%)* | 16.7 (2.9) | 16.0 (3.6) | 1.00 |
| **Disease duration, years (SD)** | 6.9 (5.3) | 6.9 (4.7) | 1.00 |
| **Levodopa use (%)** | 19 (65.5) | 29 (72.5) | 1.00 |
| **SBP change from supine to standing at 3 minutes, mmHg (SD)** | -19.9 (13.4) | -26.3 (14.1) | .72 |
| **DBP change from supine to standing at 3 minutes, mmHg (SD)** | -5.3 (9.2) | -4.0 (9.3) | 1.00 |
| **Hoehn & Yahr scale (SD)** | 2.6 (0.9) | 2.3 (0.8) | 1.00 |
| **MDS-UPDRS Part 3 (SD)** | 30.1 (17.6) | 30.9 (14.9) | 1.00 |
| **MoCA score (SD)** | 23.5 (4.8) | 23.8 (5.2) | 1.00 |
| **MCI (%)** | 9 (31.0) | 14 (35.) | 1.00 |
| **Dementia (%)** | 7 (24.1) | 8 (20.0) | 1.00 |
| Continuous variables are reported as mean (standard deviation); categorical variables are reported as number (percentage). Statistical significance marked with *. The results reported in this table are the results of Fisher’s exact test and independent t-test with Bonferroni-adjusted *p* values.  **Abbreviations:** SD: Standard deviation; MDS-UPDRS: Movement Disorders Society Unified Parkinson Disease Rating Scale; MoCA: Montreal Cognitive Assessment; MCI: Mild cognitive impairment | | | |
